# Integrated Metabolomics and Network Pharmacology Analysis Immunomodulatory Mechanisms of Qifenggubiao Granules

**DOI:** 10.3389/fphar.2022.828175

**Published:** 2022-04-05

**Authors:** Bindan Guo, Wenting Dong, Jinhai Huo, Guodong Sun, Zhiwei Qin, Xiaodong Liu, Bihai Zhang, Weiming Wang

**Affiliations:** Institute of Chinese Materia Medica, Heilongjiang Academy of Chinese Medicine Sciences, Harbin, China

**Keywords:** Qifenggubiao granules, metabonomics, network pharmacology, cyclophosphamide, immunosuppression

## Abstract

**Background:** Qifenggubiao granules (QFGBG) is a new Chinese medicine independently developed by Heilongjiang Academy of Traditional Chinese Medicine, which combines the essence of Yupingfeng powder and Shengmai yin (invention patent number: CN1325098C, approval number: Sinopharm Zhunzi B20020410), and has been included in the 2020 edition of Chinese Pharmacopoeia. It has remarkable pharmacodynamic results and conclusive clinical effects in the treatment of allergic rhinitis, chronic cough and other diseases. Previous pharmacological studies have shown that it has immunomodulatory effect, but its immunomodulatory mechanism is still unclear.

**Methods:** In this study, cyclophosphamide (CTX) was used to establish the immune hypofunction model in mice, and the weight change, index of immune organs in spleen and thymus, pathological sections of immune organs and inflammatory factors were used to evaluate the model. Based on the metabolic biomarkers obtained by metabonomics technology, the potential targets of Qifeng Gubiao Granule immunomodulation were obtained by integrating the targets of blood components, metabolites and diseases through network pharmacology. Meanwhile, GO enrichment analysis and KEGG pathway analysis were carried out on the potential targets.

**Results:** QFGBG can increase body weight and organ index, and recover immune organ damage caused by CP. Metabonomics identified 13 metabolites with significant changes, among which the level of phospholipid (PC) metabolites decreased significantly in the model group. Sphingosine -1- phosphate, 1- palmitoyl phosphatidylcholine [LysoPC (16:0/0:0)] and other metabolites were significantly increased in the model group, and 98 targets of Qifeng’s external immune regulation were obtained by intersecting 629 component targets, 202 metabolite targets and 1916 disease targets. KEGG pathway analysis obtained 233 related metabolic pathways, and the top 20 metabolic pathways mainly involved IL-17 signaling pathway, TNF signaling pathway, Sphingolipid signaling pathway, and so on.

**Conclusion:** QFGBG may act on AKT1, IL6, MAPK3, PTGS2, CASP3, MAPK1, ESR1, PPARG, HSP90AA1, PPARA and other targets, acting through Sphingolipid signaling pathway and signaling pathway. Combined with pharmacodynamic evaluation, the immunomodulatory effect of QFGBG was confirmed, and the immunomodulatory mechanism of QFGBG with multiple targets and multiple pathways was preliminarily clarified.

## Introduction

Qifenggubiao granules (QFGGB) is a new Chinese medicine independently developed by Heilongjiang Academy of Traditional Chinese Medicine (invention patent number: CN 1325098C, approval number: Sinopharm Zhunzi B20020410), which is included in Chinese Pharmacopoeia in 2020 edition, and is composed of six medicinal herbs: *Astragalus mongholicus Bunge (Fabaceae), Eleutherococcus senticosus (Rupr. & Maxim.) Maxim (Araliaceae), Atractylodes macrocephala Koidz (Asteraceae), Saposhnikovia divaricata (Turcz. ex Ledeb.) Schischk (Apiaceae), Ophiopogon japonicus (Thunb.) Ker Gawl (Asparagaceae), and Schisandra chinensis (Turcz.) Baill (Schisandraceae),* with a ration of 6:3:2:2:2:1. It combines the essence of Yupingfeng powder and Shengmai Yin, and replaces *Panax ginseng C.A.Mey (Araliaceae)* with Heilongjiang genuine medicinal material Eleutherococcus senticosus (Rupr. & Maxim.) Maxim (Araliaceae), which has the effects of tonifying lung and kidney and invigorating spleen, and is included in the 2020 edition of Chinese Pharmacopoeia (Edited by National Pharma, 2015). It mainly contains flavonoids, organic acids, polysaccharides and other components, all of which have immunoregulatory effects (Dandan et al., 2020; Ran et al., 2020), Modern pharmacological studies preliminarily show that it can improve the body immunity through improve the phagocytic function of macrophages and increase the formation rate of erythrocyte C_3b_ receptor garland (Weiming and Shuming, 2005), and restore the immune balance of allergic rhinitis (AR) rats (Tianshuai et al., 2021), but its immune regulation mechanism has not been reported yet.

In order to study the immunomodulatory mechanism of QFGBG, cyclophosphamide (CTX) was used to establish a mouse model of hypoimmunity. CP is an alkylating agent, which can destroy immune cells, interfere with the proliferation and differentiation of T and B cells, reduce the number of normal T and B cells, and inhibit cellular and humoral immune responses (Xie et al., 2015). Because of its strong immunosuppressive effect on immune-related cells (Yunbo, 2013), the immunosuppressive mouse model induced by CP has been used as a valuable tool to detect the good immunoprotective efficacy of natural compounds (Kim et al., 1211; Yoo et al., 2020).

Metabonomics studies the human body as a complete system, which has the characteristics of wholeness, dynamics, non-trauma, close to physiology, etc. It is very similar to the principle of wholeness and dynamics of traditional Chinese medicine, and provides a new and powerful technical means for traditional Chinese medicine research (Shan et al., 2015). Network pharmacology is based on the interaction between “disease-gene-target-drug,” and constructs the related network from the perspective of biological system, explores the pathogenesis and course development of diseases, and reveals the interaction between various targets of drugs in the body, which is beneficial to the multi-level exploration of the action mechanism of active compounds of traditional Chinese medicine (Bing et al., 2017). The research methods of metabonomics and network pharmacology based on the holistic view are consistent with the systematic view advocated by traditional Chinese medicine research, so they are especially suitable for studying the action mechanism of complex systems of traditional Chinese medicine (Li et al., 2021).

This study will combine metabonomics and network pharmacology methods, metabonomics identifies potential biomarkers, and at the same time, network pharmacology is used to find biomarker targets and correlate component targets and disease targets, Narrow the target range of QFGBG immunomodulation, and provide technical support for the follow-up research.

## Materials and Methods

### Experimental Instruments and Reagents

QFGBG (Produced by Pharmaceutical Factory of Heilongjiang Institute of Traditional Chinese Medicine, batch number: 07210501, which meets the standards on pages 979–980 of Chinese Pharmacopoeia I, 2020), Cyclophosphamide injection (jiangsu hengrui Pharmaceutical Co., Ltd.; batch number: 20092225), Levamisole hydrochloride tablets (Renhetang Pharmaceutical Co., Ltd.; batch number: 201001), Watsons water, Formic acid (Fisher Company), Acetonitrile and methanol (Merck, Germany), UltraSYBR One Step RT-qPCR Kit (CW0659, CoWin Biosciences, China), themouse serum IL-4 (EK0405), IFN-γ (EK0375) ELISA kit (Boster, China), Mouse ACTB Endogenous Reference Genes Primers, 10 μM (B661302-0001, Sangon Biotech, China).

Nitrogen generator (Hangzhou Dacker, DFNW-5LB), Cryogenic centrifuge (Thermofisher, 17R), Upright white photo microscope (Nikon Japan, Eclipse Ci-L), Scanning software (3 dhistech Hungary, CaseViewer2.4), Panoramic slice scanner (3DHISTECH Hungary, PANNORAMIC, DESK/MIDI/250/1000), Ultra-high performance liquid chromatograph (AB Sciex ExionLC AD, United States), Mass spectrometer (AB SCIEX Triple-TOFTM 5600+, United States), Infifinite M200 PRO (Tecan, Switzerland), Bioer Line gene 9600 flfluorescence Quantitative PCR instrument (Hangzhou Bioeri, China).

### Animal Experiment

There are 90 male ICR mice (about 26 g) with animal certificate number SCXK (Liao) 2020-0001, which are provided by Liaoning Changsheng Biotechnology Co., Ltd. The study was conducted strictly according to the ethical guidelines for using experimental animals in Heilongjiang province, guided and approved by the animal ethics committee of the academy of traditional Chinese medicine of Heilongjiang province [(2011)93]. Adaptive feeding for 7 days, ICR mice were randomly divided into 6 groups, with 15 mice in each group, they are normal control (NC), model control (MC), QFGBG low-dose(L), QFGBG medium-dose(M), QFGBG high-dose(H) and postive control levamisole hydrochloride (PC).NC group administered normal saline as well as MC, low-dose group administered QFGBG (13 g/kg), medium-dose group administered QFGBG (26 g/kg), and high-dose group administered QFGBG (52 g/kg). positive control administered levamisole hydrochloride (25 mg/kg). On the 8th day, mice in each group except the blank group were injected with CP 80 mg/kg intraperitoneally for three consecutive days, From the 11 days, each group was given corresponding doses of drugs, 15 consecutive days.

Mice in each group fasted for 12 h after the last administration time, The mice were weighed before modeling and after the experiment, and the weight changes of mice in each group were measured. and whole blood was collected, Approximately 1 mL of whole blood was collected, The serum was obtained through centrifuged at 3000 rpm and 4°C for 15 min, the spleen and thymus of each group of mice were collected and weighed, And then fixed with 4% neutral formaldehyde. The liver tissue was washed and frozen.

### Pharmacodynamic Evaluation

The fixed spleen and thymus organs were washed with distilled water and dehydrated with ethanol gradient. Xylene was transparently treated twice, soaked in wax, and embedded in a paraffifin embedding machine. The embedded tissue was fixed on a microtome for sectioning and placed on a clean carrier. On the glass slides, the slides are dried in a 60°C constant temperature oven, and then dewaxed, HE stained, dehydrated, and finally sealed with neutral gum.

### Biochemical Index Detection

After the mouse serum was thawed, it was centrifuged at 3000 rpm for 10 min. The supernatant was taken for ELISA detection, and a microplate reader was used for detection.

### Metabonomics Sample Preparation

Serum samples were thawed at 4°C before preparation. 100 μL serum samples were diluted with 300 μL methanol (precooled at 4°C), swirl at 2500 rpm for 1.5 min, Centrifuge at 4°C and 13000 rpm for 10 min blow dry the supernatant with nitrogen, and freeze at −80°C. Before running on the machine, 150 μL of 80% methanol (precooled at 4°C) was re-dissolved, and centrifuged at 4°C 13000 rpm at 4°C for 10 min. The supernatant was taken and put into the inner liner tube, and the liquid was sampled. 10 μl of serum was drawn from all samples, which were mixed and used as QC samples. QC samples were tested every 5 samples to evaluate the stability of the system.

### UPLC-Q-TOF-MS Conditions

The Waters Acquity UPLC BEH C18 column (100 mm × 2.1 mm, 1.7 μm), aquityupl-cbeh C18 vanguard pre-column (100 mm × 2.1 mm, 1.7 μm), the column temperature is 35°C, and the mobile phase is 0.1% formic acid solution (A) −0.1% formic acid acetonitrile (B). 2–5 min, 60–40% B, 5–11 min, 30–70% B, 11–13 min, 10–90% B, 13–14 min, 100% B, 14.1–17 min, 95–5% B.

ESI ion source is used, ionization mode is positive and negative ion mode, ion source voltage is 5500 V, ion source temperature is 550°C, cracking voltage is 80 V, collision energy is 40 V, CES is 20 eV, atomizing gas is N2, auxiliary gas and atomizing auxiliary gas are both 55 PSI, air curtain gas is 35 PSI, and scanning range of sub-ions is 80–1600 Da. Eight peaks with IDA response value exceeding 100 cps were set for secondary mass spectrometry scanning, and the scanning range of Produc-tIon was 80–1600 Da, and dynamic background subtraction was started. The data acquisition software is Analyst TF 1.6 software, and the image processing system is Peakview 2.0.

ESI ion source is used, ionization mode is positive and negative ion mode, ion source voltage is 5500 V, ion source temperature is 550°C, cracking voltage is 80 V, collision energy is 40 V, CES is 20 eV, atomizing gas is N2, auxiliary gas and atomizing auxiliary gas are both 55 PSI, air curtain gas is 35 PSI, and scanning range of sub-ions is 80–1600 Da. Eight peaks with IDA response value exceeding 100 cps were set for secondary mass spectrometry scanning, and the scanning range of Produc-tIon was 80–1600 Da, and dynamic background subtraction was started. The data acquisition software is Analyst TF 1.6 software, and the image processing system is Peakview 2.0.

### Network Pharmacology Research

#### Blood Components and Disease Target Prediction

Enter the SMILE format of blood component (Dandan et al., 2021) into Swiss target prediction database (http://www.swisstargetprediction.ch/). The target of the final composition is obtained after removing the duplicate target from the Database. The Online Mendelian Inheritance in Man (OMIM,https://omim.org/), The TTD (http://db.idrblab.net/ttd/), the database (TTD, http://db.idrblab.net/ttd/), Drug and Target Database (https://go.drugbank.com/), DisGeNET Database (https://www.disgenet.org/) were used to search for the immune dysregulation-related targets. Enter the target points of components into Cytoscape 3.8.2 to construct the network visualization diagram of “medicinal materials-components-targets”.

#### Prediction of Differential Metabolite Targets

Based on metabolomics, the obtained differential metabolites were input into HMDB database to obtain the SMILE format of substances and input into Swiss Target Prediction database to obtain metabolite targets. Enter the target into Cytoscape 3.8.2 to construct the “metabolite-target” network diagram.

#### Construction of Immune Regulation Network Diagram

The component target, disease target and metabolite target were input into Venny 2.1 software to obtain the intersection target, and the PPI network diagram was obtained by importing the intersection target into String (https://string-db.org/) website. Furthermore, the nodes in the network are ranked according to the Degree by using the CytoHubba plug-in of Cytoscape 3.8.2, and the top 10 targets are selected as potential targets.

#### Analysis of Related Channels

The intersection targets were input into Metpascape (http//metascape.org/) website for GO and KEGG analysis, with *p* < 0.05. GO function analysis is mainly used to describe the functions of gene products, including biological process (BP), cell composition (CC) and molecular function (MF). KEGG is used to analyze the possible action pathway of the target and to explore the mechanism of immune enhancement of QFGBG.

#### Data Processing

The data collected by UPLC-Q-TOF-MS were subjected to peak detection, peak alignment and normalization by Progenesis QI software, and then the data were imported into EZinfo software for analysis. According to the variable importance projection (VIP) value of OPLS/PLS-DA model, VIP >1, *p* < 0.05 was selected to find the differential metabolites.

GraphPad Prism version 8.0.2 software was used for all statistical analysis. All data are expressed as average ± standard deviation (‾X±SD) and the difference between the two groups was compared by *t* test, and the *p* value < 0.05 showed statistical significance.

## Results

### Pharmacodynamic Evaluation

**FIGURE 1:**
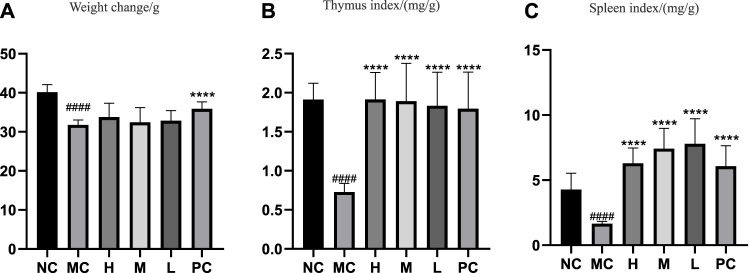
**(A)** Effects of QGGBG and CP on the weight change of ICR mouse. **(B)** Effects of QGGBG and CP on thymus index of ICR mouse. **(C)** Effects of QGGBG and CP on spleen index of ICR mouse.

**FIGURE 2:**
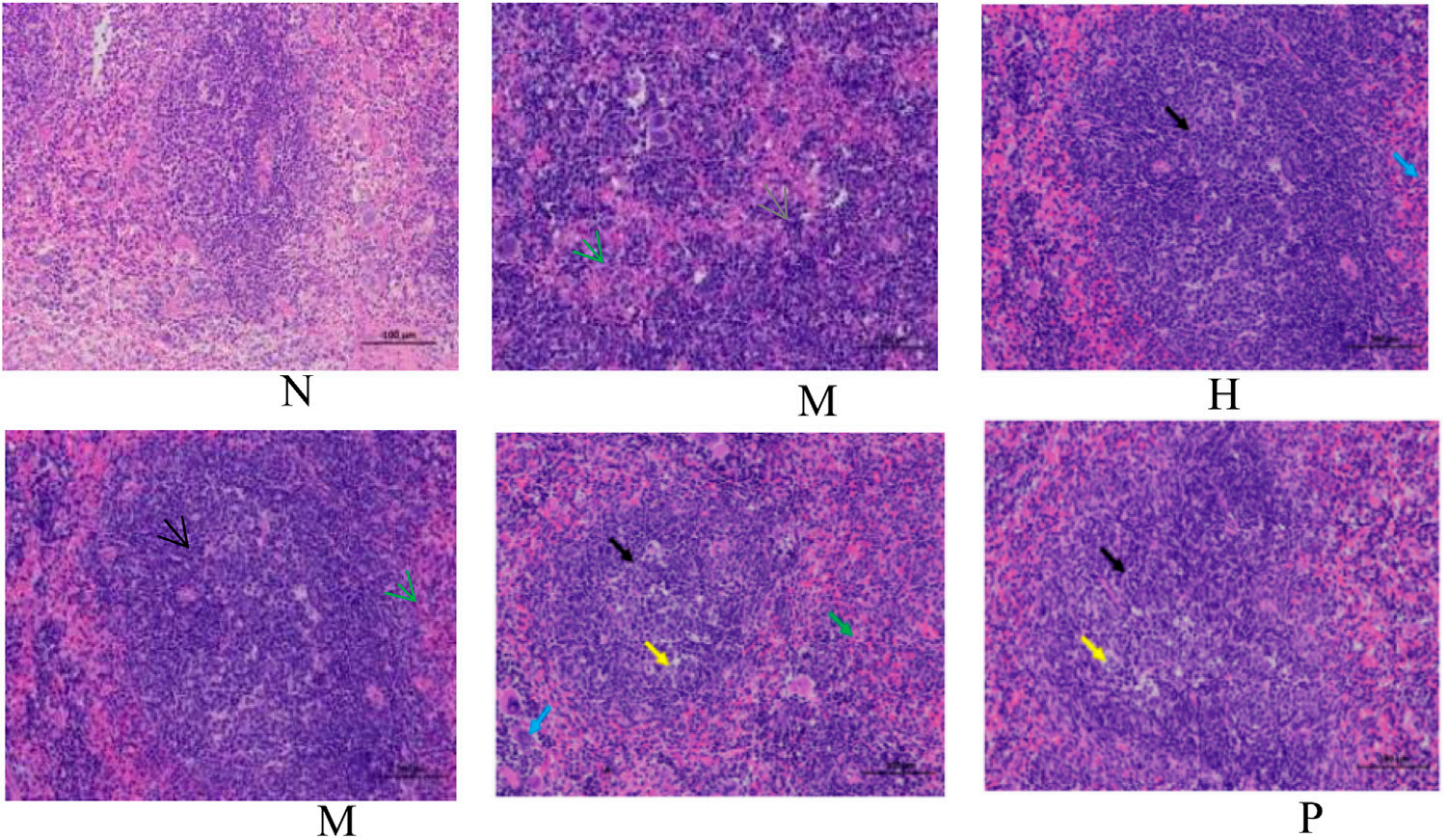
Effects of QFGBG on the spleen tissues in the HE stained histopathological images. (200×, the black arrow represents the expansion of germinal center, the yellow arrow represents nuclear fragmentation, the green arrow represents neutrophil infiltration, the blue arrow represents a small increase in the number of multinucleated giant cells, and the gray arrow represents extramedullary hematopoiesis).

**FIGURE 3:**
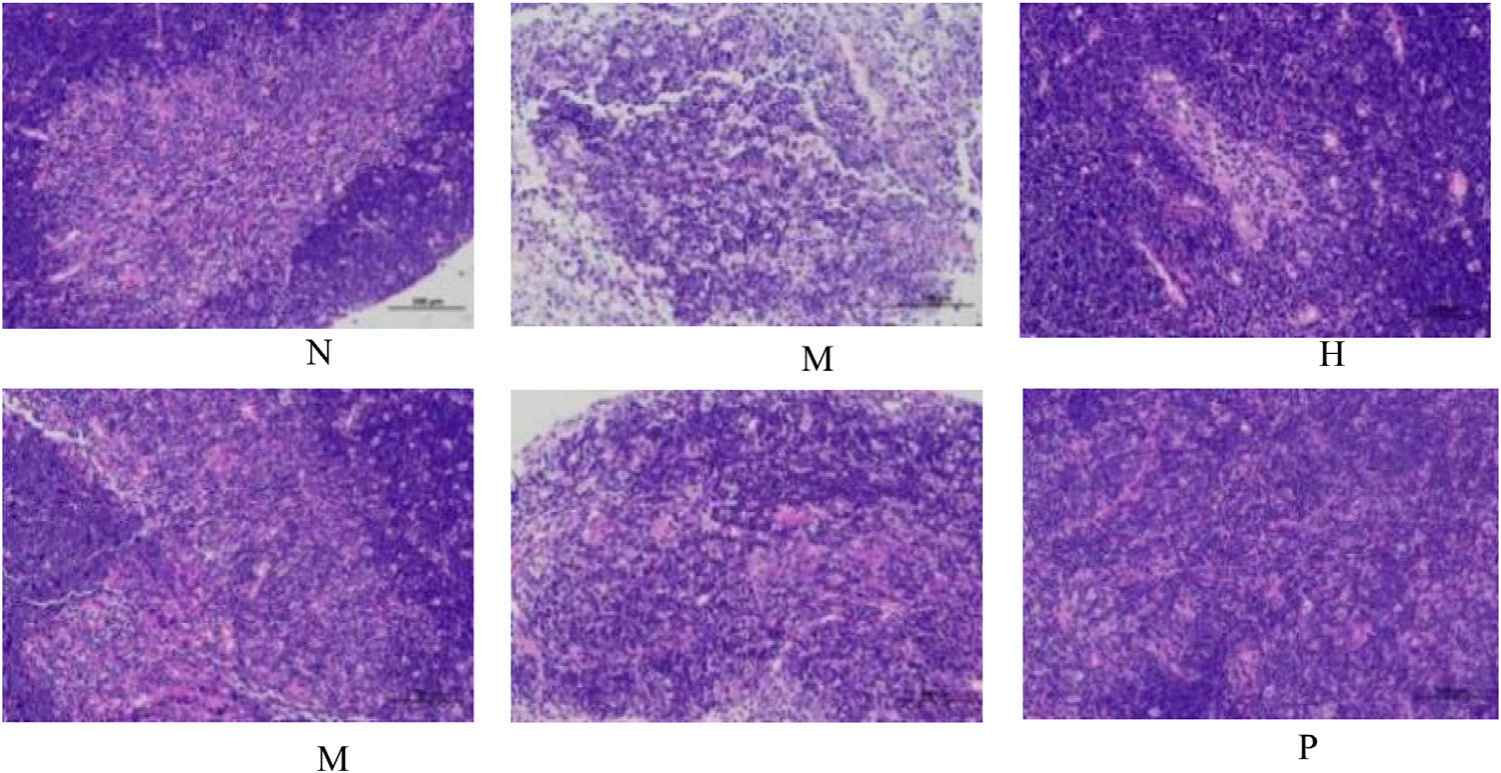
Effects of QFGBG on the thymus tissues in the HE stained histopathological images. (200×).

The change in body weight and organ index is shown in Figure 1A. It can be seen that the weight of the model group is obviously reduced compared with that of the blank group (*p* < 0.001), and all of them recovered after administration, and PC had the most obvious recovery effect (*p* < 0.001). The index changes of thymus and spleen are shown in Figures 1B,C, the spleen and thymus of the model group all showed significant atrophy, which is significant difference with the blank group (*p* < 0.001), and both showed significant recovery after administration (*p* < 0.001). HE staining of spleen and thymus is shown in Figures 2, 3, In the model group, the number of spleen nodules decreased significantly, extramedullary hematopoiesis was found in the red pulp (black arrow), neutrophil infiltration was rare (green arrow), and the germinal center was enlarged in the blank group (black arrow), which indicated that inflammatory reaction occurred, and lymphoid tissue produced protective response to the body, after administration, the number of spleen nodules basically recovered, neutrophil infiltration (green arrow), a small amount of nuclear fragmentation (yellow arrow) and a small increase in the number of multinucleated giant cells (gray arrow) were rare, and there was no significant difference between the rest and the blank group. Thymic cells were sparse, and the boundaries between cortex and medulla were blurred, indicating that immune organs were damaged. After administration, all patients were adjusted back, and there was no significant difference among the groups. Thymus and spleen are important immune organs of the body. The size of thymus index can reflect the immune status of the body. From the above results, it can be concluded that the immune function of mice is inhibited, and QFGBG has a certain effect on improving immunity.

### Biochemical Index Determination

**FIGURE 4:**
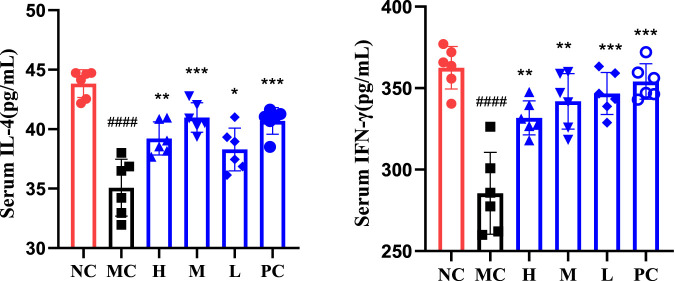
Serum IL-4 and IFN-γ expression levels. The significant difference was calculated using analysis of variance, # *p*-values of NC vs. MC, ####*p* < 0.0001, ###*p* < 0.001, ##*p* < 0.01, #*p* < 0.05,* *p*-values compared with MC, ****p < 0.0001, ***p < 0.001, **p < 0.01, *p < 0.05.

Using IFN-γ, IL-4 as evaluation indicators Figure 4. T lymphocytes play an important role in the body’s immunity, among which CD4 + T cells can be expressed on the surface of helper T cells (Th), which can be divided into Th1 cells and Th2 cells, among which Th1 cells mainly secrete cytokines such as IL-2 and IFN-γ, and their levels can reflect the degree of inflammation damage, while Th2 cells mainly secrete cytokines such as IL-4 and IL-10, which are closely related to the progress and chronic diseases (Borish and Rosenwasser, 1996; Hirata et al., 2001; Wynn Thomas, 2015). IFN-γ is a cytokine with various biological activities produced by activated T lymphocytes, and it is an important immunomodulatory factor *in vivo* (Manjunatha et al., 2012), On the one hand, it can resist virus, inhibit virus replication and proliferation, and on the other hand, it can activate macrophages, strengthen antigen presentation process and enhance cellular immune function of organism (Panpan, 2014). IL-4 is an anti-inflammatory cytokine, which can reflect the immune function of the body to a certain extent. The results showed that compared with the NC, the levels of IFN-γ and IL-4 in the MC were significantly reduced, indicating that the immune response of mice was suppressed, and the contents of each group were increased to varying degrees after administration, indicating that QFGBG could effectively improve the self-repair function of injured immune cells and further promote the recovery of the immune function of the body.

### Metabonomics Results

#### PCA and (O)PLS-DA Analysis

**FIGURE 5:**
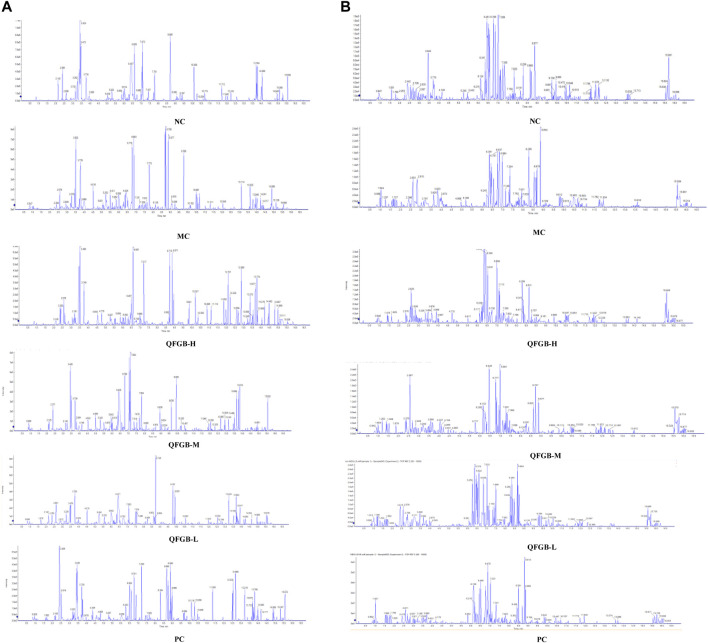
The TIC of six group. **(A)** ESI+; **(B)** ESI−.

**FIGURE 6:**
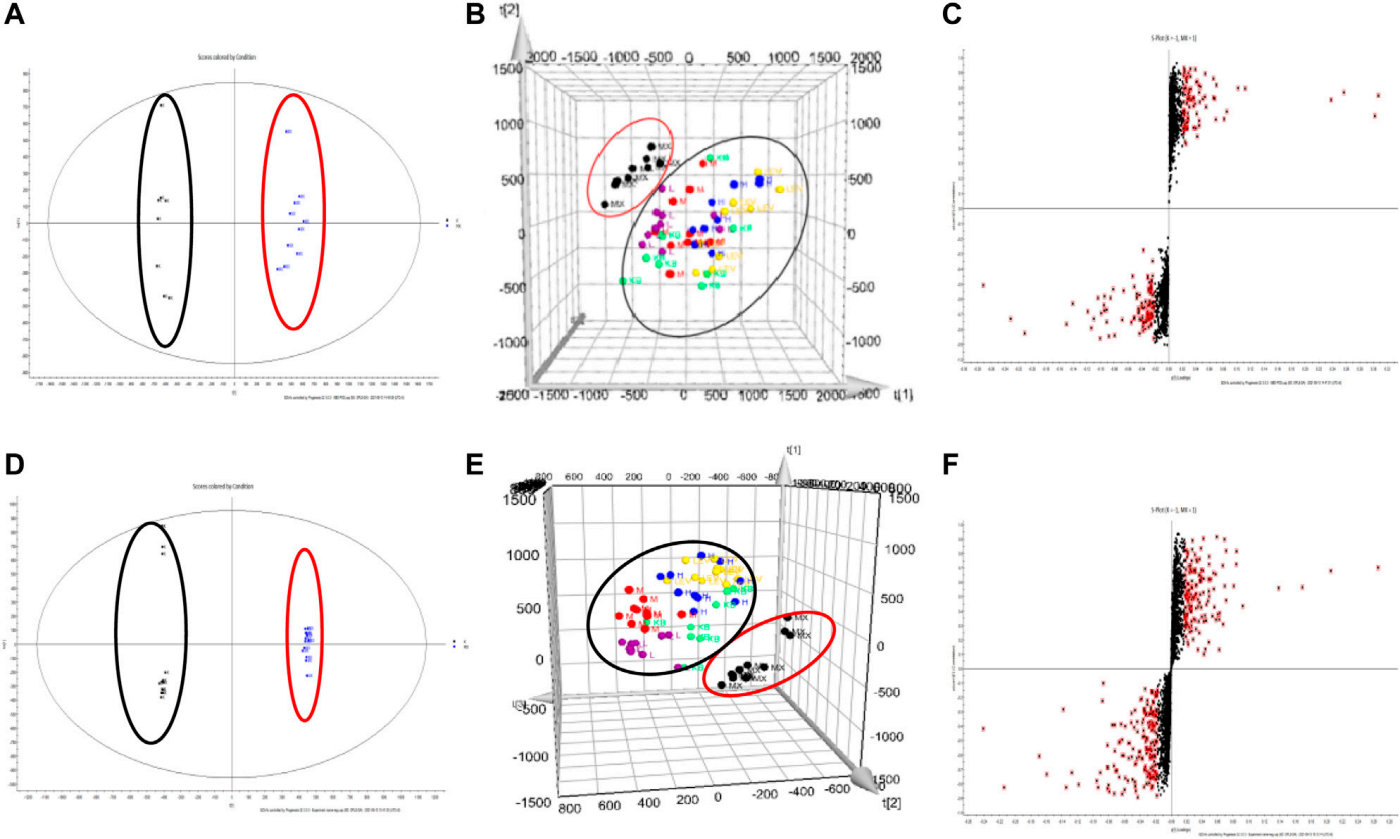
**(A,D)** represents respectively the OPLS-DA two-dimensional graph of NC and MC (red stands for NC and black stands for MC) **(B,E)** represents respectively the PLS-DA three-dimensional diagram of the six group **(C,F)** represents respectively the S-plot graph of NC and MC **(A–C)** stands for ESI+; **(D–F)** stands for ESI−.

UPLC-Q-TOF/MS was used to analyze the metabonomics of serum samples from six groups of mice. The TIC diagram is shown in Figure 5. There is no obvious difference in each group from TIC diagram, which needs further analysis. The results of metabonomics analysis of each group are shown in Figure 6. From blank and model OPLS-DA two-dimensional diagrams (Figures 6A,D), it can be seen that the clustering of the two groups clearly indicates that PC causes the changes of metabolites in mice, and the metabolites of each group approach the blank after administration (Figures 6B,E), which indicates that QFGBG has a callback effect on the changes of metabolites caused by CP.

#### 3.3.2 Determination and Analysis of Significant Differences

**FIGURE 7:**
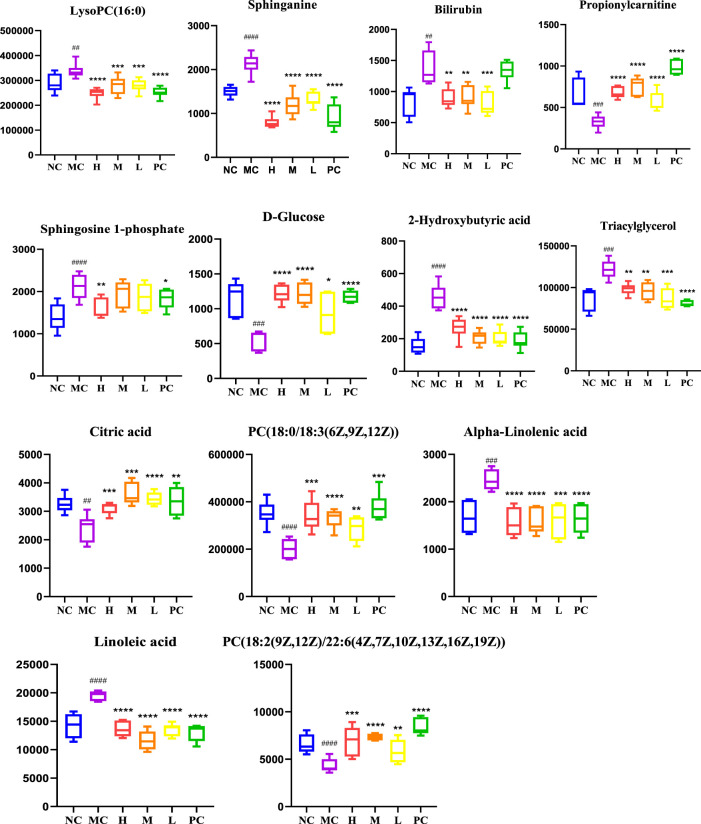
Changes in levels of the potential biomarkers in the six groups. # *p*-values compared withMC; ####*p* < 0.0001, ###*p* < 0.001, ##*p* < 0.01, * *p*-values compared with MC, ****p < 0.0001, ***p < 0.001, **p < 0.01, *p < 0.05.

In order to identify the potential biomarkers of QFGBG playing an immunomodulatory role from thousands of variables, the variables were uniformly extracted with *p* < 0.05 and VIP >1 as parameters in combination with S-Plot (Figures 6C,F) and OPLS-DA diagrams and matched with HMDB (http://www.hmdb.Ca) online database, and finally 13 differential markers were determine (Table 1). Mainly lipids (glycerophospholipids, sphingolipids, glycerides) and some fatty acid metabolites (linoleic acid, α-linolenic acid), in which the levels of phospholipids (PC), glucose and citric acid metabolites were significantly reduced in the model group; Sphingosine -1- phosphate, 1- palmitoyl phosphatidylcholine [LysoPC(16:0/0:0)], bilirubin, linoleic acid and other metabolites increased in the model group, and QFGBG could adjust the contents of these metabolites. Taking the abundance of 13 biomarkers as ordinate and groups as abscissa, we can clearly see the differences among groups (Figure 7).

**TABLE 1:** Potential biomarkers of immunedys regulation.

No.	Retention time (min)	m/z	HMDB ID	Molecular formula	MS/MS	Mass error (ppm)	Metabolites	VIP	Trend	Adducts
1	7.047116667	496.3391024	HMDB10382	C24H50NO7P	496.3398;478.3292;184.0733;166.0628;104.1070	−1.339657256	LysoPC(16:0)	12.8004	↑##	M + H
2	5.29415	302.304604	HMDB00269	C18H39NO2	302.3042;284.2948;106.0863;88.0757;57.0699	−2.495755536	Sphinganine	2.1454	↑####	M + H
3	3.150766667	585.270166	HMDB00054	C33H36N4O6	585.2708;568.2443;550.2337;412.1867;285.1234;267.1129	−1.019001737	Bilirubin	1.48879	↑##	M + H
4	1.8212	218.1374324	HMDB00824	C10H19NO4	218.1387;159.0652;144.1020;57.0335	−5.766686337	Propionylcarnitine	1.02012	↓###	M + H
5	0.94125	203.0512566	HMDB00122	C6H12O6	203.0526;145.0496;85.0285;57.0335	−7.509597353	D-Glucose	1.04127	↓###	M + Na
6	11.71848333	784.58456	HMDB08040	C44H82NO8P	784.5851;725.4991;601.5191;184.0733;60.0808	−0.665788027	PC(18:0/18:3(6Z,9Z,12Z))	12.9616	↓####	M + H
7	8.697666667	1047.737099	HMDB10561	C69H100O6	1047.7238;525.3614;524.3672;184.0730	−4.013308875	Triacylglycerol	7.96941	↑###	M + Na
8	15.53698333	830.5678648	HMDB08156	C48H80NO8P	852.5514/830.5524;771.4960;669.4853;184.0733;86.0965	−1.888676062	PC(18:2(9Z,12Z)/22:6(4Z,7Z,10Z,13Z,16Z,19Z))	2.45112	↓####	M + H, M + Na
9	5.851483333	380.2554225	HMDB00277	C18H38NO5P	380.2561;364.2611;284.2948;264.2686;120.9661	−1.618790293	Sphingosine-1-phosphate	1.62037	↑####	M + H, M + Na
10	1.737733333	103.0406311	HMDB00008	C4H8O3	103.0437;73.0325;59.0502;57;0346;	5.415183325	2-Hydroxybutyric acid	1.01357	↑####	M − H
11	1.524233333	191.0193906	HMDB00094	C6H8O7	191.0197;173.0091;154.9986;129.0193;115.0036;72.9931	−1.747673614	Citric acid	2.2793	↓##	M − H
12	9.435466667	277.2173996	HMDB01388	C18H30O2	277.2183;259.2067;233.2274;182.1312;71.0138	0.344590538	Alpha-Linolenic acid	2.19489	↑###	M − H
13	10.47606667	279.2334667	HMDB00673	C18H32O2	279.2329;261.2224;205.1962;149.0972;83.0502	1.830270977	Linoleic acid	5.2674	↑####	M − H

a Change trends compared with the normal. The levels of differential metabolites were marked with downregulated (↓) and upregulated (↑).

Notes: #*p*-values of NC vs. MC, ####*p* < 0.0001, ###*p* < 0.001, ##*p* < 0.01.

### Network Pharmacology Results

#### Prediction of Target Points of Blood Components of Qifenggubiao Granules

**FIGURE 8:**
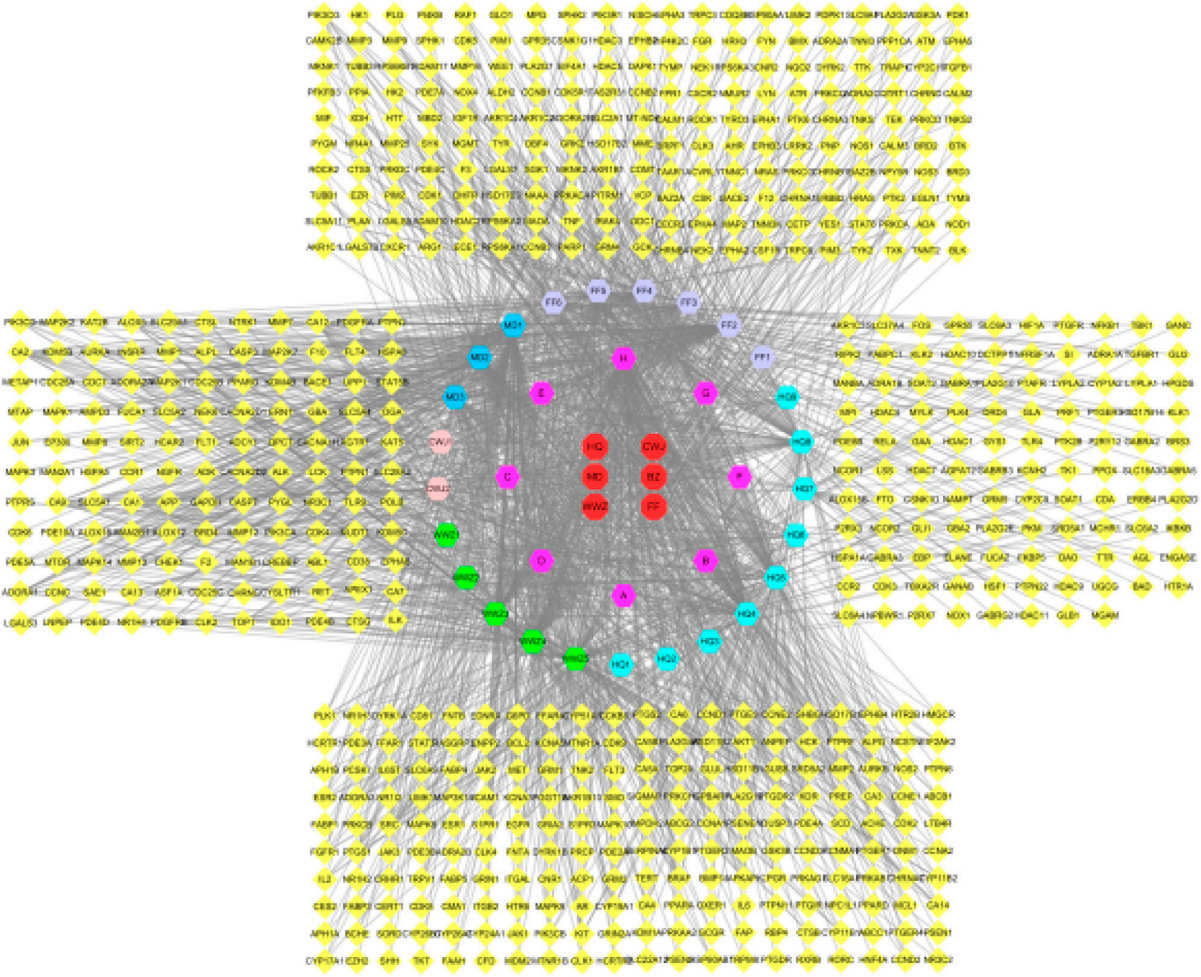
Interaction network of components and targets of QFGBG (yellow represents the target and other colors represent the components).

A total of 33 blood components and 629 component targets were identified, and the target points were input into the software of Cytoscape3.8.2 to obtain the “component-target” (Figure 8). As shown in the Figure 8, yellow represents the target and other colors represent the components.

#### 3.4.2 Prediction of Metabolite Targets of Qifenggubiao Granules

**FIGURE 9:**
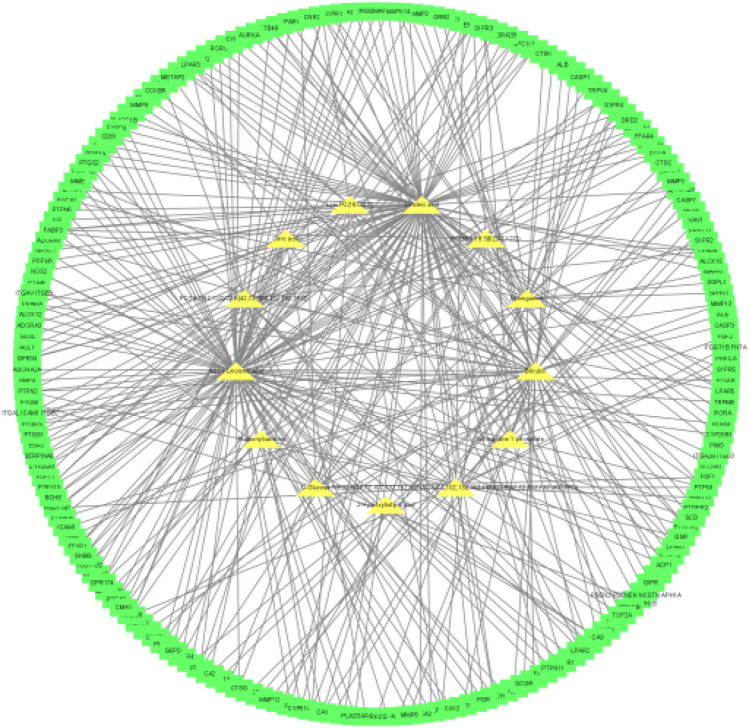
Interaction network of components metabolite and targets of QFGBG (Green squares represent targets, and yellow triangles represent metabolites).

A total of 13 metabolic biomarkers and 202 metabolite targets were identified, and the “metabolite-target” is shown in (Figure 9). Among them, yellow triangles represent metabolites and green rectangles represent targets.

#### Analysis of Immune Regulation Target Protein Network of Qifenggubiao Granules

**FIGURE 10:**
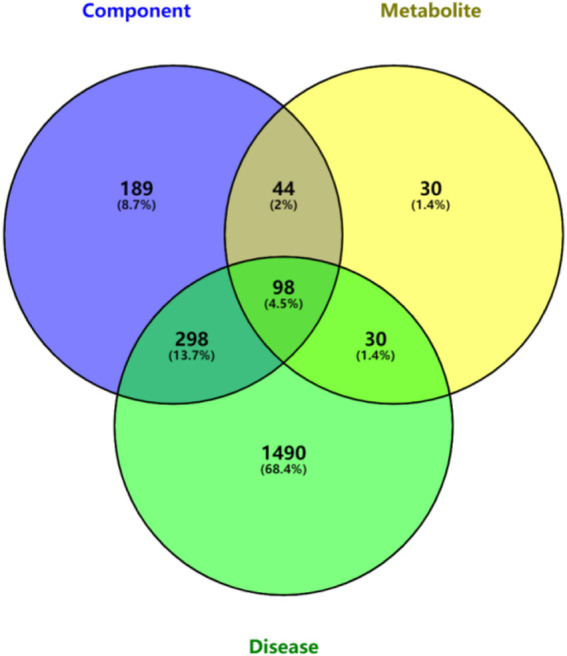
Components, metabolites, disease intersection target venn diagram.

**FIGURE 11:**
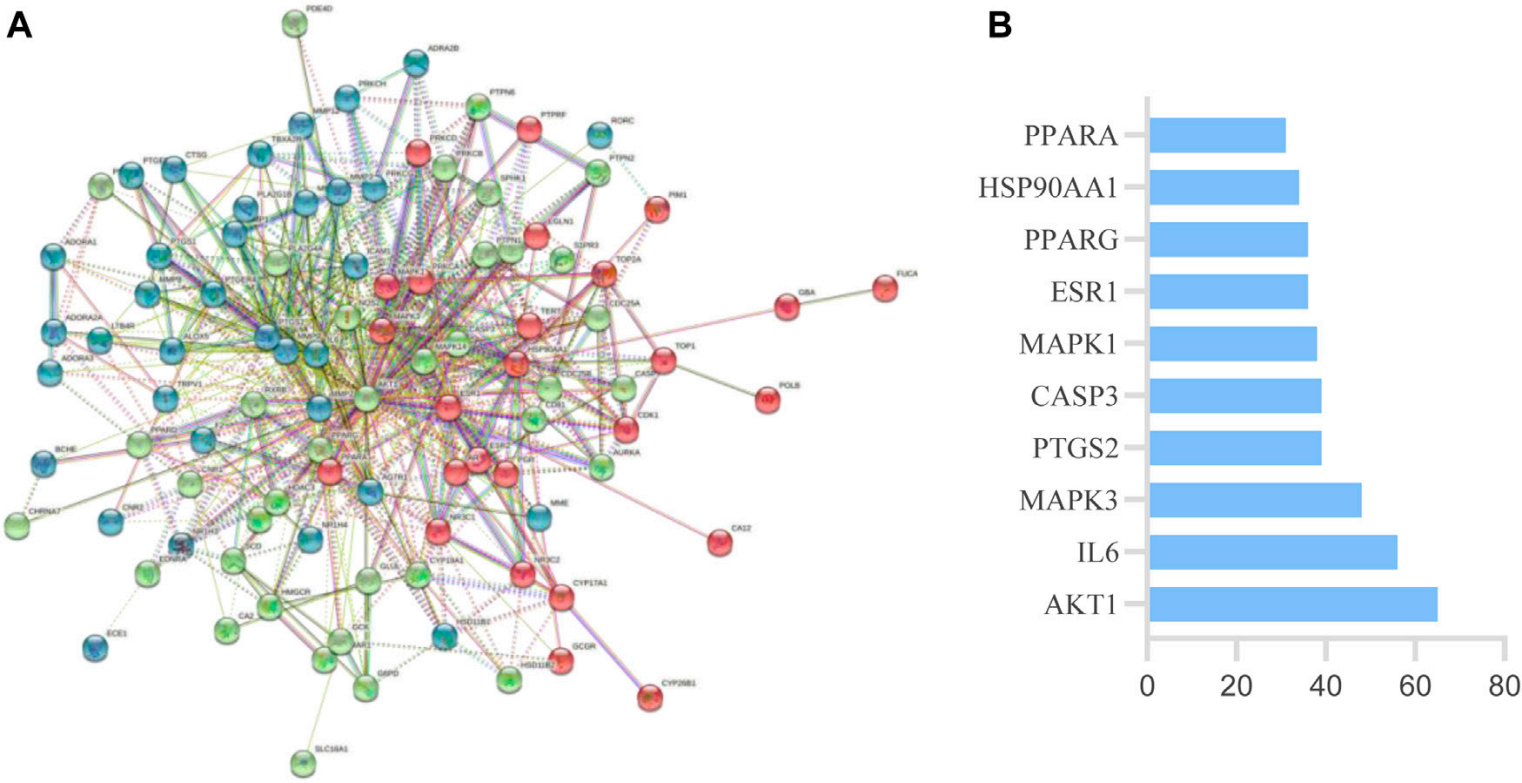
**(A)** Protein-protein interaction network of mutual target. **(B)** The top 10 target of PPI.

A total of 135 intersection targets were obtained from 629 component targets, 202 metabolite targets and 1916 disease targets of QFGBG, and 98 (Figure 10) intersection targets were input into String database to construct PPI network diagram, as shown in (Figure 11A). The minimum required interaction score is 0.4, the PPI network graph has 98 nodes and 634 edges, and the average degree of nodes is 12.9. Each node represents a protein, and the connection between nodes represents the interaction between protein. The common target may be related to the immune regulation mechanism of QFGBG. The network diagram of protein interaction is helpful to understand the functional relationship between protein more intuitively. Entering intersection targets into Cytoscape. First, the targets whose degree is greater than the mean (12.9) in the network are screened. On this basis, the first 10 nodes whose degree is greater than the mean degree (degree >24.4857142857143) are defined as the core targets (Figure 11B).

#### GO Enrichment Analysis and KEGG Pathway Analysis

**FIGURE 12:**
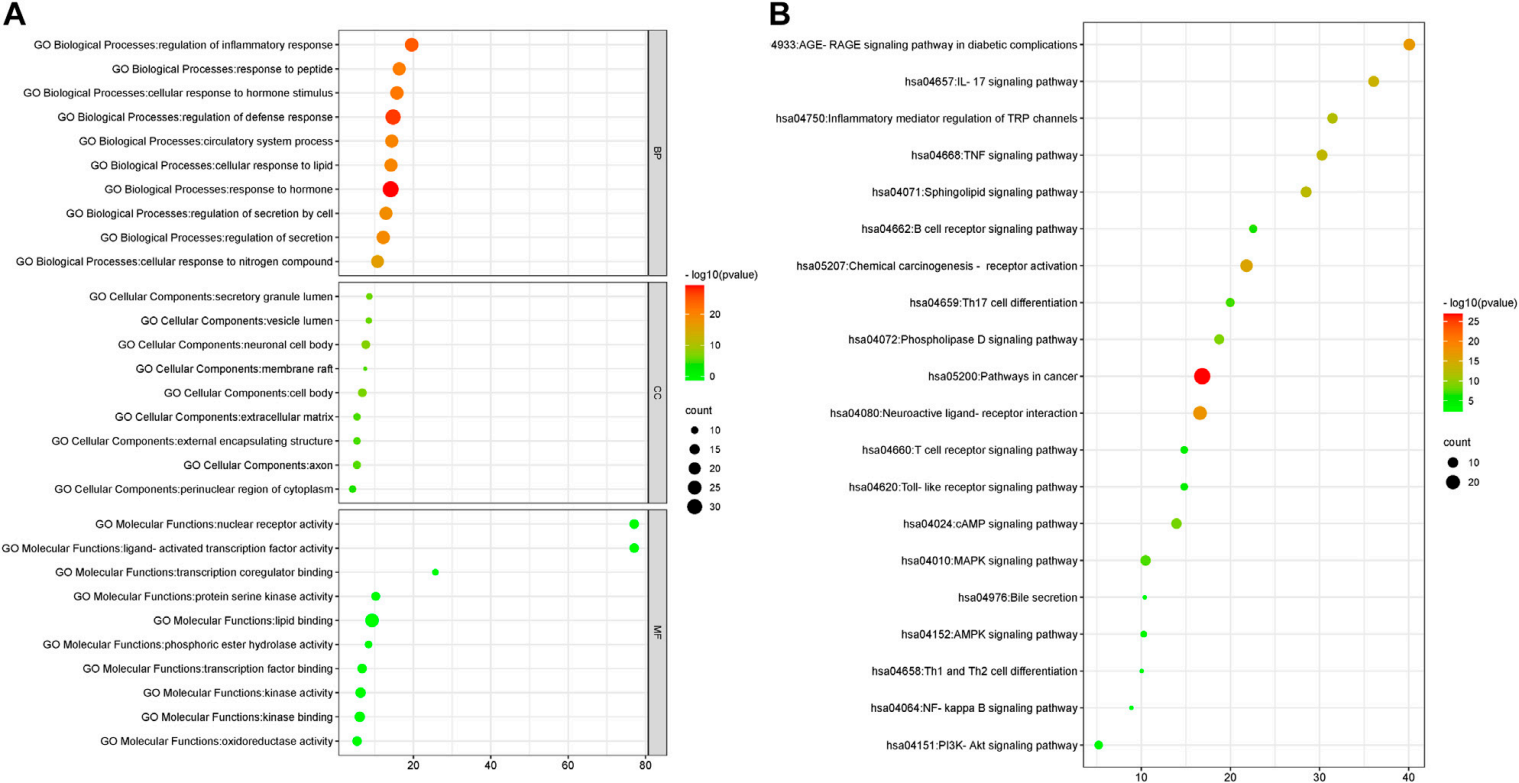
**(A)** GO gene enrichment function analysis diagram. **(B)**: KEGG pathway enrichment analysis diagram (abscissa represent the number of targets in the signaling pathway, bubble size represents the number of genes in the pathway; the ordinate represents the signal pathway; bubble color represents the *p* value, the red color, the larger *p* value).

To further reveal the immune regulation mechanism of QFGBG, we used Metscape database to analyze GO enrichment and KEGG pathway of the above 98 targets. GO analysis mainly includes three parts, biological process (BP), cellular component (CC), and molecular function (MF). GO analysis showed that BP included 1139 enrichment results, including regulation of defense response, regulation of inflammatory response, regulation of secretion. CC includes 76 enrichment results, mainly involving neuronal cell body, external encapsulating structure, cell body, and secretory granule lumen. The MF includes 108 enrichment results, including lipid binding, nuclear receptor activity, protein kinase activity etc. The enrichment results are sorted according to *p* value. The online analysis platform (http://www.bioinformatics.com.cn) is used to draw the top 10 analysis results of three parts, as shown in (Figure 12A). 233 pathways were obtained by analysis of KEGG gene annotation functional analysis. These pathways mainly include Sphingolipid signaling pathway, Inflammatory mediator regulation of trpchannels, MAPK signaling pathway etc. The results of visual analysis of the top 20 pathways are shown in (Figure 12B).

## Discussion

**FIGURE 13:**
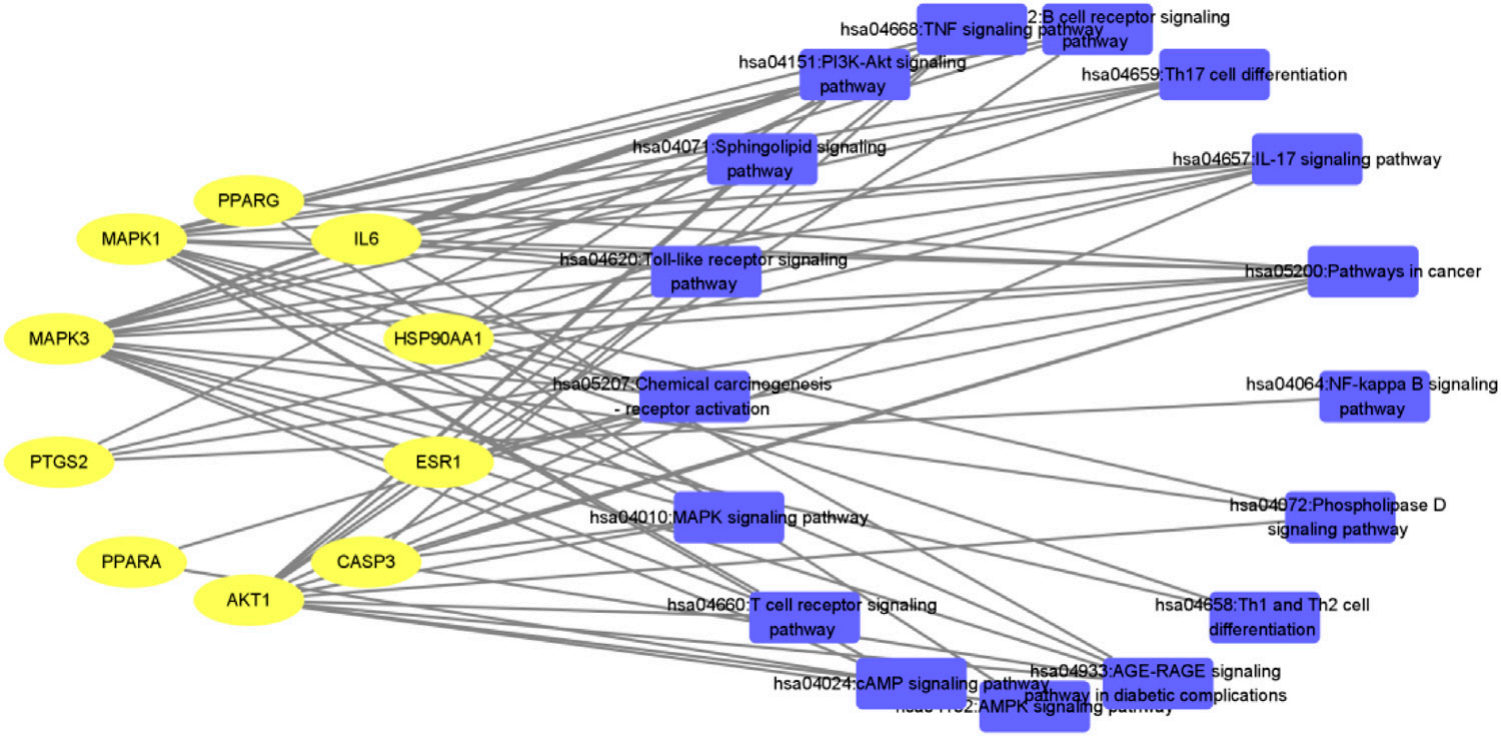
Interaction network of “target-pathway” of QFGBG (yellow ellipses represent the target site, and blue rectangles represent the pathway).

**FIGURE 14:**
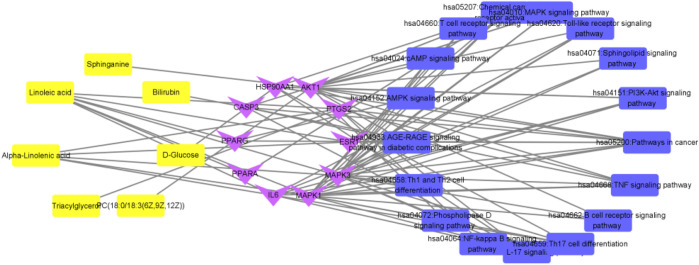
Interaction network of “metabolite-target-pathway” of QFGBG (yellow represents metabolites, purple represents targets, and blue represents pathways).

Immunity is a physiological function of the human body. The human body relies on this function to identify “self” and “non-self” components, thus destroying and rejecting antigens entering the human body or damaged cells and tumor cells produced by itself, so as to maintain human health. The immune system mainly has three functions: defense, stabilization, and surveillance. Once these functions are out of balance, pathological reactions will occur. Inflammation is a manifestation of immune response to infection or injury, and is the result of activation of innate and/or acquired immune response (June 2017), that is, inflammation is a manifestation of initiation and activation of immune response (June 2019). Immunoregulation involves multi-target and multi-pathway, which is a complex mechanism of interaction between immune cells, immune molecules, immune organs, and other systems (such as neuroendocrine system, etc.). Traditional Chinese medicine has complex components, and there is synergistic effect among multiple components, metabonomics, and network pharmacology are based on the balance of system biology and biological network. Starting from the overall model, they can improve or adjust the balance of biological network by adjusting signal pathways in multiple ways to improve the curative effect of drugs (Qi, 2018), which is more in line with the holistic view and systematization of traditional Chinese medicine. The results showed that QFGBG could reverse the damage of immune organs caused by CTX and increase the levels of IL-4 and IFN-γ in serum, Metabonomics analysis shows that QFGBG can increase the level of phospholipid (PC) metabolites and reduce the contents of sphingosine, sphingosine -1- phosphate, triglyceride, and other metabolites. Furthermore, 98 possible immunomodulatory targets of Qifeng solid surface can be obtained by network pharmacological analysis. AKT1, IL6, MAPK3, PTGS2, CASP3, MAPK1, ESR1, PPARG, HSP90AA1, and PPARA are the key targets of immune regulation. The KEGG pathway analysis of potential targets shows that QGGBG may play an immunomodulatory role through inflammatory pathways such as inflammatory mediator regulation of Trp channels, Sphingolipid signaling pathway, and MAPK signaling pathway. The key targets obtained from network pharmacology are enriched into the same or different metabolic pathways, and then the levels of various metabolites are regulated to regulate the whole organism. See Figures 13, 14 for the specific visualization diagrams of “target-pathway” and “target-metabolite-pathway,” respectively.

### Sphingolipid Signaling Pathway

Studies have shown that cyclophosphamide can cause liver injury (Huanying, 2019). Liver plays a key role in metabolism, biosynthesis, secretion, and detoxification. In recent years, it has been found that sphingolipid metabolism is involved in the occurrence and development of liver diseases. Sphinganine is not only an important component of cell membrane structure, but also an inactive precursor in sphingolipid metabolism and an important effector molecule in cell signal transduction (Jiuli et al., 2011). Tumor necrosis factor (TNF-α) is most related to the pathological damage process of liver, which can affect the proliferation, growth, inflammation, regeneration, and apoptosis of liver cells (Shaoyuan et al., 2015). TNF-α can activate the activities of sphingosine kinase 1 and sphingosine kinase 2(SPHKs), which in turn catalyzes the phosphorylation of Sphinganine to produce S1P (Yanhong et al., 2019). However, high concentration of S1P can mediate the internalization of S1P receptors (S1PRs) and reduce the outward migration of lymphocytes in the immune system, thus achieving the immunosuppressive effect (Yoshihiko et al., 2016), the contents of sphingosine and S1P in the model group increased significantly (*p* < 0.001), which indicated that Sphinganine might be phosphorylated to S1P by SPHKs catalysis, and then play an immunosuppressive role. After administration, the content of each group increased. It shows that QFGBG can play an immunomodulatory role by reducing the contents of Sphinganine and S1P through sphingolipid signaling pathway.

### Mitogen-Activated Protein Kinases Signaling Pathway

Mitogen-activated protein kinases (MAPKs) are a kind of serine/threonine protein kinases, which widely exist in mammalian cells. MAPK pathway is an important way to transfer extracellular signals into cells and cause cell biological reactions (such as cell proliferation, differentiation, transformation, and apoptosis, etc) (Yanwu, 2016), which mainly includes three cascade reactions. Extracellular signal-regulated protein kinase (ERK) pathway, c-jun N-terminal kinase (JNK) pathway and p38 MAPK pathway, the activation of which can promote the production of inflammatory cytokines and aggravate inflammatory reaction (Seger and Krebs, 1995). Modern pharmacological studies have shown that MAPK is closely related to liver diseases, respiratory diseases and other diseases (Jinchan et al., 2022; Zhihua et al., 2020; Tianyi et al., 2021). Studies have shown that reducing ERK, p38 MAPK mRNA expression and p-ERK and p-p38 MAPK expression in rat lung tissue can improve airway inflammation in asthmatic rats (Feng et al., 2018). Dephosphorylation of p38 MAPK can not only alleviate excessive lung inflammation induced by macrophage and PMNs recruitment, but also inhibit macrophage from non-inflammatory apoptosis to inflammation (Li et al., 2018). Phosphatidylcholine (PC) is not only an important component of biofilm system in eukaryotes, but also a signal molecule of cell signal transduction (Exton, 1994). Changes in PC content and structure can lead to abnormal enzyme activity, receptor function and membrane permeability, thus affecting metabolism and function of organisms (Caiyun, 2008). Lysophosphatidylcholine (LysoPC) is one of the metabolites of mammalian phospholipids and the hydrolysis product of phospholipase A2 of phosphatidylcholine, which is widely involved in many physiological and pathological processes including inflammatory reactions (Sedlis et al., 1993). When the biofilm is damaged, MAPK, as an important cell conduction pathway, will inevitably be affected. In the experiment, the content of PC in CP group decreased significantly, and LysoPC increased, indicating that its biofilm was damaged. After administration, the contents of PC and LysoPC in each group were adjusted back to different degrees, indicating that QFGBG can regulate the content of downstream PC through MAPK, and then play an immunomodulatory role.

### Inflammatory Mediator Regulation of Transient Receptor Potential Channels

Inflammation plays an important role in many kinds of diseases, which mainly refers to various inflammatory diseases caused by complex reactions caused by tissue injury or pathogen infection (Xin et al., 2019). TRP channel is a kind of ion channel, which can be activated by heat, and TRPV1~4 are typical thermosensitive transient receptor potential channels (Holzer, 2011). They will be stimulated by exogenous or endogenous substances, and produce various neuropeptides, which will lead to neurogenic inflammation such as local tissue vasodilation and increased permeability, inflammatory cell exudation, mucosal congestion and edema, and bronchoconstriction (Grace et al., 2014). And the high expression of TRPAl/TRPV1 plays an important role in the pathogenesis of chronic cough (Bonvini et al., 2015; Bonvini and Belvisi, 2017; Xinyang and Shunan, 2014). This is consistent with the clinical direction of QFGBG, and on the other hand, it is explained that QFGBG may play a role in the treatment of chronic cough through TRP channel.

## Conclusion

Network pharmacology provides key technical support for revealing the scientific connotation of traditional Chinese medicine compound, discovering drug targets and guiding the research and development of new traditional Chinese medicine drugs (Wenxia et al., 2012). Traditional network pharmacology studies mostly combine chemical components and disease targets to study traditional Chinese medicine. From the perspective of system level and biological network as a whole, it analyzes the law of molecular association between drugs and therapeutic objects (World Traditional Chinese, 2021), The entry of traditional Chinese medicine into the blood may be the material basis of its efficacy (Chunlu et al., 2020), Metabolites are considered to be the most intuitive reflection of physiological and pathological conditions of organism, and subtle changes of gene expression and protein expression will be amplified on metabolites (XiJun, 2015), and key metabolites can provide reference for disease phenotype (Xiongjian et al., 2021), Metabonomics, through the judgment and further analysis of metabolites, finally gets a whole result (Weiwei et al., 2011). Metabonomics explores the regulatory mechanism of gene function, providing reliable basis for drug treatment or disease pathogenesis. Network pharmacology reveals the pathogenesis of complex diseases and the therapeutic mechanism of drugs from the system level, which are integrated and complementary to each other (Wang, 2020), And the immune regulation mechanism of QFGBG was studied from gene level and metabolite level. In this study, we found that CP caused immunosuppression in mice. QFGBG can improve the organ index of mice in each group, repair the pathological damage caused by CP and play an immunomodulatory role. According to the results of network pharmacology and metabonomics, QFGBG may act on AKT1, IL6, MAPK3, PTGS2, CASP3, MAPK1, ESR1, PPARG, HSP90AA1, PPARA, etc. Through Sphingolipid signaling pathway, MAPK signaling pathway, inflammatory mediator regulation of trp channels further regulate the content of metabolites such as PC, LysoPC, Sphinganine, etc. and play an immunomodulatory role. The above results indicate that QFGBG plays a multi-target, multi-channel, multi-level, and multi-faceted role in immune regulation.

## Data Availability

The original contributions presented in the study are included in the article/Supplementary Material, further inquiries can be directed to the corresponding authors.
